# Orchestrating mitochondrial protein import: The emerging role of DYRK1A in health and disease

**DOI:** 10.1002/pro.70631

**Published:** 2026-05-25

**Authors:** Sahana Shankar, Chris Meisinger

**Affiliations:** ^1^ Institute of Biochemistry and Molecular Biology, ZBMZ, Faculty of Medicine University of Freiburg Freiburg Germany; ^2^ CIBSS—Centre for Integrative Biological Signalling Studies University of Freiburg Freiburg Germany

**Keywords:** *Down syndrome*, DYRK1A, *DYRK1A‐related syndrome*, mitochondrial protein import, organellar signaling, TOM complex

## Abstract

The translocase of the outer mitochondrial membrane (TOM complex) serves as the central entry gate for more than 1000 nuclear‐encoded precursor proteins imported into the organelle. Recently, the human import receptor TOM70 has been identified as a substrate of the serine/threonine kinase DYRK1A. DYRK1A activates the metabolite carrier import pathway, and its impairment triggers a transcriptional adaptive response that induces remodeling of the TOM complex. This compensatory mechanism activates additional import pathways to mitigate reduced DYRK1A signaling. Patients with dysfunctional DYRK1A signaling exhibit clinical manifestations that resemble classical features of mitochondriopathies. The emerging *DYRK1A‐TOM70* axis therefore represents a central signaling platform coordinating mitochondrial protein import pathways in health and disease.

## 1 INTRODUCTION

The mitochondrial proteome comprises approximately 1400 different proteins in humans and about 1000 proteins in yeast (Meisinger et al., 2008; Pagliarini et al., 2008). Only a small fraction of these proteins is encoded by the mitochondrial genome and synthesized within the organellar matrix (13 in humans and eight in yeast), whereas the majority of the proteome is built via import of nuclear‐encoded precursor proteins from the cytosol. Several import and sorting machineries have evolved to mediate translocation of preproteins across the two mitochondrial membranes as well as sorting into one of the four mitochondrial subcompartments—outer membrane, intermembrane space, inner membrane, and matrix—after membrane translocation. This process depends on the presence and characteristics of distinct targeting signals (presequences) within the precursor protein (Busch et al., 2023; Chacinska et al., 2009; Neupert & Herrmann, 2007; Vögtle et al., 2009). These protein biogenesis machineries enable mitochondria to establish and maintain their proteome and thereby ensure a functional organelle capable of fulfilling its numerous cellular roles in health and disease (Calvo & Mootha, 2010; Harbauer, Opalińska, et al., 2014; Meichsner et al., 2026; Nunnari & Suomalainen, 2012; Song et al., 2021).

**FIGURE 1:**
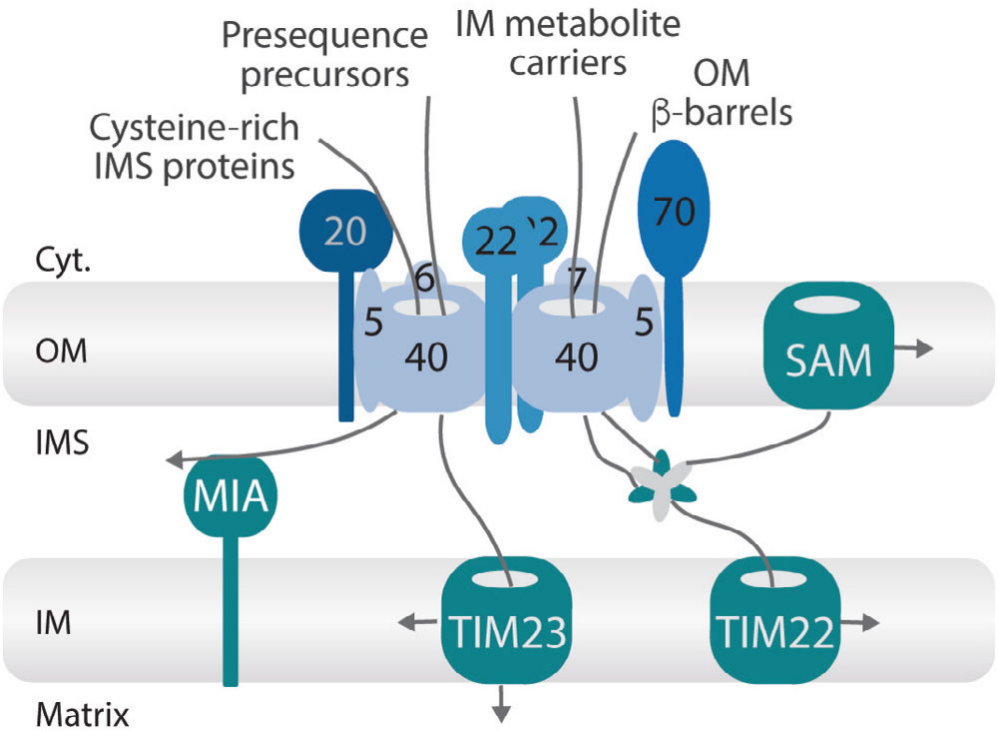
Overview of mitochondrial protein import pathways highlighting the central role of the translocase of the outer mitochondrial membrane (TOM) complex as the main entry gate for different classes of precursor proteins. Numbers indicate individual TOM subunits. SAM, sorting and assembly machinery for β‐barrel precursor proteins in the outer membrane (OM). MIA, import of precursor proteins containing characteristic cysteine motifs, which are retained in the intermembrane space (IMS) through oxidative folding mediated by the MIA pathway. TIM23, translocase responsible for the import of N‐terminal presequence‐containing precursor proteins into the matrix or into the inner membrane (for single‐pass transmembrane proteins). TIM22, translocase mediating the insertion of polytopic carrier precursor proteins into the inner membrane after their chaperone‐assisted transfer across the IMS by small TIM proteins.

The translocase of the outer membrane (TOM complex) is the central entry gate for nearly all precursor proteins from the cytosol. From here, precursors are further sorted, for example, to the matrix via the TIM23 translocase or to the intermembrane space via the MIA machinery. Members of the metabolite carrier family are chaperoned across the intermembrane space via small TIM proteins and then inserted into the inner membrane via the TIM22 translocase (Busch et al., 2023; Chacinska et al., 2009; Chatzi et al., 2016; Endo & Yamano, 2009; Neupert & Herrmann, 2007; Riemer et al., 2011; Schneider, 2020; Schulz et al., 2015; Figure 1).

The TOM complex consists of a core complex comprising the central pore‐forming subunit Tom40, the central import receptor Tom22, and three small subunits, Tom5, Tom6, and Tom7. Moreover, Tom20 and Tom70 serve as import receptors for the two major classes of preproteins: while Tom20 preferentially recognizes precursor proteins containing N‐terminal presequences, Tom70 binds precursors of the metabolite carrier family, which are hydrophobic inner membrane proteins delivered to Tom70 with the assistance of cytosolic chaperones. Tom70 binds, unlike Tom20, internal targeting sequences that are less well characterized (Backes et al., 2018; Brix et al., 1999). Both, Tom20 and Tom70 can dynamically associate with the core TOM complex in order to deliver the precursor cargo to the central receptor Tom22 and the translocation pore Tom40 for import (Meisinger et al., 2001). It has also been reported that Tom20 and Tom70 can to some extent have overlapping substrate specificity. Tom70 has also been proposed to have chaperone‐like activities (Backes et al., 2018; Brix et al., 1999; Kreimendahl & Rassow, 2020; Yamamoto et al., 2009). Given the central role of Tom70 in the biogenesis of metabolite carriers and its dynamic association with the TOM core complex, modulation of Tom70 activity represents a particularly effective point of control for regulating mitochondrial protein biogenesis.

While the TOM complex has long been considered a rather static machinery that serves as a constitutively active gate for incoming precursor proteins, several studies in the model organisms yeast and mouse have identified cytosolic protein kinases that target the TOM complex and thereby control, for example, import receptor function, stability, or biogenesis of this machinery (Gerbeth et al., 2013; Harbauer, Zahedi, et al., 2014; Kravic et al., 2018; Schmidt et al., 2011). These studies have led to a new view of a dynamic and regulated import machinery that is connected to, for example, changes in cellular metabolism or to the cell cycle phase (see detailed reviews in Harbauer, Opalińska, et al., 2014; Matic et al., 2018; Opalińska & Meisinger, 2015). However, whether the human TOM complex might also be a target of cytosolic protein kinases remained unclear until recently.

## 2 DISCOVERY OF DYRK1A AS THE FIRST HUMAN TOM KINASE

While the yeast kinome, with approximately 110 different kinases, is relatively manageable, the human kinome comprises more than 500 kinases, making the identification of kinases targeting specific human proteins considerably more complex (Edwards et al., 2011). To search for putative kinase candidates targeting the human TOM complex, Walter et al. (2021) employed the *KinaseFinder* platform (Butkevich et al., 2016), which enables high‐throughput screening of more than 350 different human protein kinases in a radiometric filter‐binding assay using a purified target protein. This approach allowed an entirely unbiased search for novel kinases acting on TOM proteins.

When the human TOM70 receptor domain was used as substrate, two members of the dual‐specificity tyrosine‐regulated kinase family, DYRK1A and DYRK1B, emerged as the top hits. Prior to this study, neither kinase had been linked to mitochondrial function. DYRK1A and DYRK1B are closely related homologs; DYRK1A is ubiquitously expressed and localized predominantly in the nucleus and cytosol, whereas DYRK1B is expressed in a more restricted set of cell types. DYRK kinases are Ser/Thr kinases that are activated through autophosphorylation of a tyrosine residue within their activation loop. For DYRK1A, several cytosolic and nuclear substrates have been identified, with important roles in processes such as cell proliferation and differentiation (Chen et al., 2013; de Lagran et al., 2012; Deboever et al., 2022; Himpel et al., 2001; Najas et al., 2015; Ryoo et al., 2007; Soppa & Becker, 2015; Tejedor & Hämmerle, 2011). The physiological role of DYRK1B is less well characterized.

**FIGURE 2:**
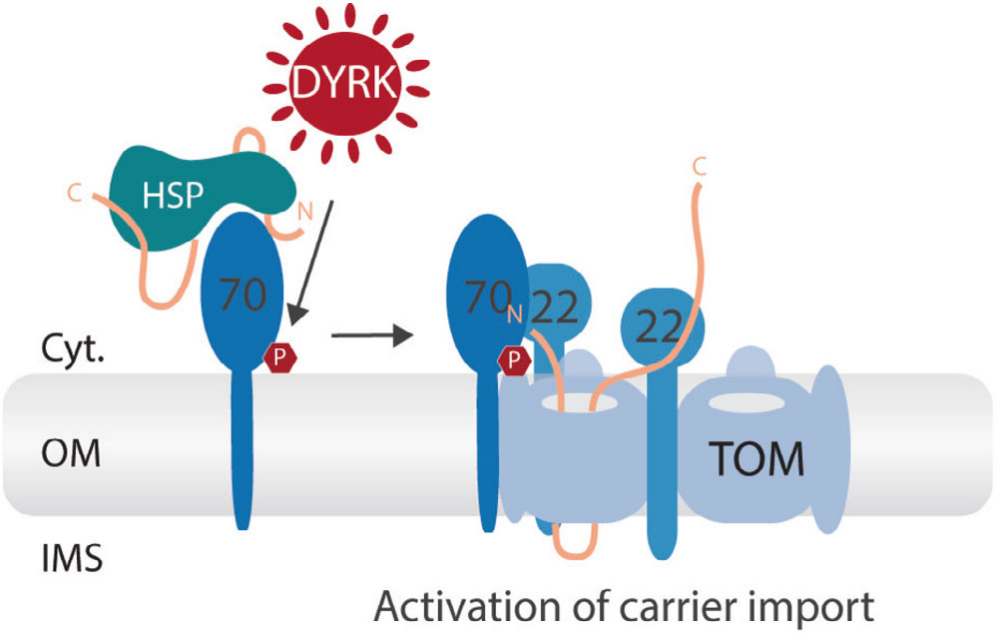
Activation of the metabolite carrier import pathway by DYRK1A. DYRK1A‐dependent phosphorylation of the import receptor TOM70 at Ser91 promotes docking of the precursor‐loaded receptor to the translocase of the outer mitochondrial membrane (TOM) core complex, thereby enhancing the import of metabolite carrier precursors into mitochondria. HSP, heat shock proteins that maintain hydrophobic precursor proteins in an import‐competent state in the cytosol until docking to the receptor; IMS, intermembrane space; OM, outer membrane.

Subsequent detailed in vitro and in vivo analyses identified Ser91 of human TOM70 as the specific phosphorylation site targeted by DYRK1A/B. It was demonstrated that phosphorylation of TOM70 at Ser91 plays a critical role in activating the import of metabolite carrier precursor proteins, which are bona fide substrates of the TOM70 receptor. Ser91 is located in close proximity to the transmembrane domain of TOM70 and appeared to be nearly quantitatively phosphorylated under all conditions tested (Walter et al., 2021). At the molecular level, phosphorylation of TOM70 at Ser91 facilitates the association of precursor‐loaded TOM70 with the TOM core complex, thereby stimulating the transfer of carrier precursors to the translocation pore and enhancing their import (Figure 2).

Around the same time, Latorre‐Muro et al. (2021) reported that TOM70^Ser91^ (and the homologous TOM70^Ser94^ in mouse) is a target of casein kinase II (CKII) and proposed that phosphorylation at this site specifically inhibits the import of the intermembrane space protein MIC19, while carrier precursor import appeared unaffected. MIC19 resides in the intermembrane space and associates with the MICOS complex of the inner membrane, which is essential for shaping mitochondrial cristae architecture (Kozjak‐Pavlovic, 2017; Rampelt et al., 2017; Sakowska et al., 2015). It was proposed that under cold stress or β‐adrenergic stimulation in brown adipose tissue, increased MIC19 import would enhance MICOS complex assembly and ultimately stimulate mitochondrial respiration. Although MIC19 is known to utilize the MIA pathway for import into the intermembrane space, it had not previously been established that MIC19 requires TOM70 as an import receptor, in contrast to metabolite carrier precursors.

This model was subsequently re‐examined in a follow‐up study from the authors of the initial identification of the *DYRK1A‐TOM70 axis* (Marada et al., 2024; Walter et al., 2021), which employed the same brown adipose tissue cell system used by Latorre‐Muro et al. (2021). In this study, β‐adrenergic stimulation did not increase MIC19 import or MICOS complex formation. Moreover, it was demonstrated that CKII does not phosphorylate TOM70, whereas DYRK1A‐mediated phosphorylation of TOM70 was independently confirmed. In addition, MIC19 was shown to depend on TOM20, rather than TOM70, as its primary import receptor. These findings argue against the existence of a *CKII‐TOM70* signaling axis in the regulation of MIC19 import and MICOS biogenesis in brown adipose tissue.

How can two entirely different protein kinases, CKII and DYRK1A, be identified for the exact same phosphorylation site? Latorre‐Muro et al. (2021) base their study on a publication that identified the homologous phosphorylation site in mouse (TOM70^Ser94^) using a high‐throughput approach. In that study, a cell extract from beige adipocytes was treated with a thermosensitive phosphatase to remove all phosphorylation sites, followed by reincubation with CKII (after thermal inactivation of the phosphatase). All phosphorylation sites subsequently detected by mass spectrometry were considered CKII‐dependent (Shinoda et al., 2015). It is highly likely that this qualitative approach may have identified unspecific sites, particularly if the phosphatase treatment was incomplete, for highly abundant phosphorylation sites such as TOM70^Ser94^. Notably, no profiling prior to CKII treatment was performed. A co‐senior author of this study later published a quantitative profiling approach that included non‐kinase‐treated controls and no longer identified TOM70 as a CKII target (Sugiyama et al., 2019). Remarkably, Latorre‐Muro et al. (2021) referred only to the earlier study and did not provide their own experimental data confirming TOM70 as a CKII target. In contrast, DYRK1A was validated not only by mass spectrometry of DYRK1A‐treated mitochondria (with and without kinase treatment), but also through biochemical approaches, including shifts in PhosTag electrophoresis and the use of phospho‐specific antisera against human TOM70^pSer91^ and mouse TOM70^pSer94^. Moreover, purified TOM70 and TOM70^Ser91Ala^ variants were used to demonstrate specificity for DYRK1A, whereas CKII failed to phosphorylate mouse or human TOM70 under the same conditions (Marada et al., 2024; Walter et al., 2021). The discrepancy between the proposed *DYRK1A‐TOM70* and *CKII‐TOM70* axes highlights the importance of validating mass spectrometry results with additional experimental approaches.

In summary, TOM70^Ser91^ represents a phosphorylation site targeted by DYRK1A/B kinases and appears to be constitutively phosphorylated. Its functional role is to promote activation of the metabolite carrier import pathway.

## 3 DYRK1A ORCHESTRATES THE MITOCHONDRIAL PROTEIN IMPORT PATHWAYS

A central approach for analyzing the physiological role of protein kinases is the application of specific inhibitors in vivo to interfere with the respective signaling pathway. Using this strategy, Walter et al. (2021) observed that inhibition of DYRK1A activity with the selective inhibitor INDY led to a transcriptional upregulation of the three import receptors TOM20, TOM22, and TOM70. In addition, DYRK1B and further members of the DYRK kinase family were also upregulated. This response appeared to represent a compensatory mechanism, suggesting that interference with the *DYRK1A‐TOM70* axis triggers a rapid remodeling of the TOM complex in order to maintain mitochondrial protein biogenesis, while simultaneously inducing DYRK1B as a potential backup kinase.

Consistent with this interpretation, all major import pathways—including the presequence pathway and the MIA pathway—were activated under these conditions. The molecular mechanisms underlying this adaptive response remain unknown. Importantly, however, this compensatory remodeling provides a mechanistic framework to reconcile the seemingly contradictory findings reported by Latorre‐Muro et al. (2021) and Walter et al. (2021).

A key experiment supporting the proposed inhibitory role of CKII‐dependent TOM70 phosphorylation on MIC19 import was the use of the CKII inhibitor CX4945 (Latorre‐Muro et al., 2021). Treatment of cultured cells with this compound led to the reported increase in MIC19 import, a finding that was also confirmed by Marada et al. (2024). However, subsequent studies demonstrated that CX4945 exhibits significant off‐target inhibition of DYRK1A (Grygier et al., 2023; Lindberg et al., 2023). Marada et al. therefore showed that treatment with CX4945 results in inhibition of DYRK1A‐dependent phosphorylation of TOM70^Ser91^, which in turn activates the previously discovered transcriptional program, including upregulation of TOM20—the primary import receptor for MIC19. This provides a mechanistic explanation for how CX4945 treatment can indirectly enhance MIC19 import.

Taken together, these findings position DYRK1A as a central regulator orchestrating multiple mitochondrial protein import pathways by coordinating TOM70 phosphorylation with adaptive remodeling of the import machinery.

Interestingly, CX4945 is currently being evaluated in clinical studies as a therapeutic CKII inhibitor for the treatment of COVID‐19 and various cancers (D'Amore et al., 2020; Naik et al., 2022). The observed interference of CX4945 with the *DYRK1A‐TOM70* axis underscores the clinical relevance of considering potential off‐target effects on DYRK1A, as unintended inhibition of this kinase may impair mitochondrial function through perturbation of mitochondrial protein import pathways.

## 4 PATHOLOGICAL IMPLICATIONS OF THE *DYRK1A‐TOM70* AXIS IN *DOWN SYNDROME* AND *DYRK1A‐RELATED SYNDROME*

Impaired DYRK1A function has been linked to a broad spectrum of clinical manifestations, including intellectual disability, short stature, delayed speech development, motor impairments, autism spectrum disorders, heart defects, epilepsy, and microcephaly, collectively referred to as *DYRK1A‐related syndrome* (Bronicki et al., 2015; Courcet et al., 2012; Earl et al., 2017; Ji et al., 2015; Luco et al., 2016; Park et al., 2009; van Bon et al., 2011, 2015; Wegiel et al., 2011; Yamamoto et al., 2011). This constellation of symptoms, particularly when occurring in combination, bears a striking resemblance to classical mitochondriopathies, which also present as multisystemic disorders (Nunnari & Suomalainen, 2012; Palmieri, 2008; Zeviani, 2001). Although DYRK1A dysfunction can cause diverse cellular defects, for example, in transcriptional regulation or cell cycle control, it is conceivable that mitochondrial function is also affected.

In light of its recently established role as a central regulator of mitochondrial protein import pathways, it is plausible that *DYRK1A‐related syndrome* may, at least in part, represent a disorder of impaired mitochondrial protein biogenesis. Interestingly, several recent studies have linked dysfunctional DYRK1A signaling to mitochondrial metabolism in cancer. For example, in a kinome‐based CRISPR screen aimed at identifying genes sensitizing cells to OXPHOS dysfunction, DYRK1A loss‐of‐function displayed a synergistic interaction with OXPHOS inhibition in hepatocellular carcinoma, leading to activation of TGF‐β signaling and enhanced anti‐tumor effects (Cao et al., 2025). These findings suggest that DYRK1A may contribute to maintaining metabolic balance in tumor cells while limiting toxicity in healthy tissues. In addition, pharmacological suppression of DYRK1A reduced both oxidative phosphorylation and glycolysis, thereby promoting metabolic reprogramming and induction of autophagy in colorectal cancer cell lines (Hwang et al., 2022). How far mitochondrial metabolism and function are indeed affected in *DYRK1A‐related syndrome* and causative of the observed symptoms needs to be clarified in the future.

Notably, DYRK1A is located within the *Down syndrome* critical region on chromosome 21 and is regarded as a key contributor to the cognitive deficits observed in individuals with Down syndrome. Due to its chromosomal location, DYRK1A is a dosage‐sensitive gene. A 1.5‐fold increase in DYRK1A gene dosage, resulting from trisomy 21, contributes to the development of *Down syndrome*. *Down syndrome* is characterized by learning and behavioral deficits, craniofacial abnormalities, early‐onset neurodegeneration, and congenital heart defects (Arron et al., 2006; Duchon & Herault, 2016; García‐Cerro et al., 2014; Kurabayashi et al., 2015). Importantly, *Down syndrome* has recently been associated with impaired mitochondrial morphology and bioenergetics (Tan et al., 2023). Similarly, proteomic profiling of the cerebellum in mice overexpressing DYRK1A to model *Down syndrome* revealed a dysfunctional mitochondrial proteome, including alterations in OXPHOS components (Ortega et al., 2022). Moreover, DYRK1A has been identified as a causative gene for congenital heart defects in *Down syndrome*: restoration of DYRK1A copy number in a mouse model improved cellular proliferation, enhanced mitochondrial respiration, and rescued heart septation defects (Lana‐Elola et al., 2024).

**FIGURE 3:**
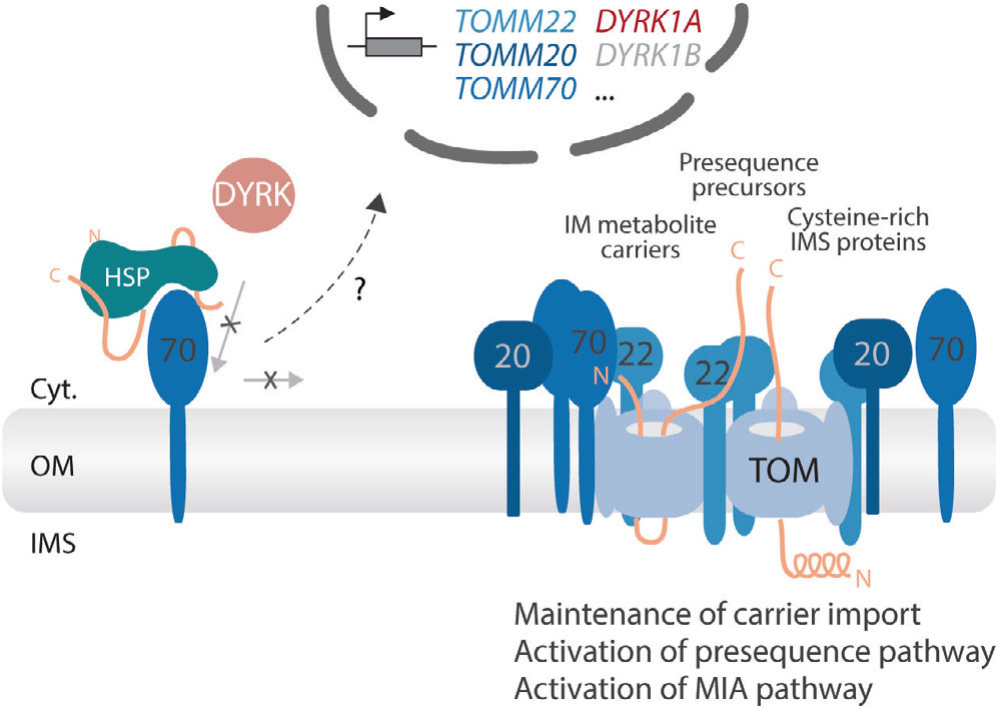
Impaired phosphorylation of TOM70 by DYRK1A triggers a compensatory transcriptional response that leads to remodeling of the translocase of the outer mitochondrial membrane (TOM) complex. Increased levels of import receptor TOM70 compensate for reduced docking efficiency at the TOM complex, thereby maintaining carrier import. Overexpression of TOM20 and TOM22 additionally activates the presequence and MIA import pathways. HSP, heat shock proteins; IMS, intermembrane space; OM, outer membrane.

These observations raise the possibility that DYRK1A overexpression in *Down syndrome* and the associated mitochondrial abnormalities may be mechanistically linked via the *DYRK1A‐TOM70* axis. However, this poses an apparent paradox: how could overexpression of a cytosolic kinase that activates the carrier import pathway result in impaired mitochondrial function? The observation that inhibition of DYRK1A triggers a transcriptional upregulation of all major import receptors—including activation of the presequence and MIA import pathways—suggests that DYRK1A gene dosage may act as a highly sensitive regulator of mitochondrial protein biogenesis in both directions, activation and suppression. Thus, not only loss‐of‐function but also excessive DYRK1A activity may disturb the delicate balance between distinct mitochondrial import pathways. Overexpression of DYRK1A could therefore lead to mitochondrial dysfunction through an imbalance in the coordinated regulation of carrier, presequence, and MIA‐dependent import routes or generally down‐regulate the import receptors transcriptionally in a converse mode as their activation by DYRK1A inhibition (Figure 3). In an alternative scenario, an overactivation of the *DYRK1A‐TOM70* axis may cause a misassembly directly at the TOM complex as excessive TOM70 phosphorylation may prevent docking of TOM20 receptors or cause its clogging via increased carrier load.

Taken together, the *DYRK1A‐TOM70* axis emerges as a central signaling platform orchestrating mitochondrial protein import pathways. While phosphorylation of TOM70 at Ser91 directly activates the carrier import pathway, impaired DYRK1A activity induces remodeling of the TOM complex and compensatory activation of the presequence and MIA import pathways while maintaining carrier import. Both insufficient and excessive DYRK1A activity therefore pose a risk to the finely tuned equilibrium of mitochondrial protein import, either through loss‐of‐function mutations causing DYRK1A‐related syndrome or through increased gene dosage in Down syndrome.

## AUTHOR CONTRIBUTIONS

**Chris Meisinger:** Conceptualization; writing – original draft; writing – review and editing; supervision; funding acquisition; project administration. **Sahana Shankar:** Conceptualization; writing – original draft; writing – review and editing.

## Data Availability

Data sharing not applicable to this article as no datasets were generated or analyzed during the current study.
